# Aripiprazole as augmentation therapy in bipolar patients with current minor or subsyndromal mood symptoms

**DOI:** 10.1186/2194-7511-1-4

**Published:** 2013-04-17

**Authors:** Isaac Schweitzer, Jerome Sarris, Virginia Tuckwell, Kay Maguire, Deidre Smith, Chee Ng

**Affiliations:** 1 Department of Psychiatry, University of Melbourne, The Melbourne Clinic, 130 Church St, Richmond, 3121 Australia; 2 Centre for Human Psychopharmacology, Swinburne University of Technology, Melbourne, 3122 Australia

**Keywords:** Bipolar disorder, Bipolar depression, Aripiprazole, Atypical antipsychotics, Adjunctive, Augmentation therapy

## Abstract

**Background:**

This study aims to evaluate the effectiveness of aripiprazole augmentation of maintenance treatment for bipolar disorder in patients with minor or subsyndromal mood episodes while on a stable dose of a mood stabiliser and/or antidepressant.

**Methods:**

All subjects had a diagnosis of bipolar I or II disorder (Diagnostic and Statistical Manual of Mental Disorders-4th Edition, Text Revision). Open-label aripiprazole was given over 8 weeks initially. The starting dose was 5 to 15 mg/day with a mean final dose of 11.5 mg (±4.6). Patients were assessed at weeks 0, 2, 4 and 8 with the Montgomery-Asberg Depression Rating Scale (MADRS), Young Mania Rating Scale (YMRS) and Clinical Global Impression of Severity (CGI-S).

**Results and discussion:**

Seventeen of 20 (85%) patients completed week 4, while 14 (70%) patients completed 8 weeks. For intention-to-treat data, there was a significant decrease in MADRS scores over the course of treatment, with a reduction of 6.40 points at endpoint (*p* < 0.0005). Improvement from baseline was significant at week 2 and remained through to week 8. Similarly, CGI-S scores significantly decreased over the course of study, but not YMRS scores. Aripiprazole was shown to be a modestly effective augmentation therapy for depressive symptoms in bipolar I and II in this small open-label study.

## Background

Increasingly, patients suffering from bipolar disorder are being treated with atypical antipsychotics (AAP). Most evidence available for their use stems from monotherapy studies of bipolar I patients who are either experiencing a manic or major depressive episode. In practice, AAPs are most often used in combination with the more traditional mood stabilisers as the majority of bipolar patients respond inadequately to monotherapy alone. Cruz et al. (2012) systematically reviewed published or registered double-blind randomised controlled trials (RCTs) using modern antipsychotics in bipolar I/II depressive patients. They identified five RCTs; four involved antipsychotic monotherapy and the other used both monotherapy and combination with an antidepressant. They concluded that only quetiapine and olanzapine have demonstrated efficacy in bipolar depressive patients, with this effect occurring rapidly from week 1 onwards. Studies exploring the use of AAPs in rapid cycling bipolar disorder are lacking, and their efficacy to stabilise mood via maintenance therapy has not yet been demonstrated (Zupancic 2011).

In a recent report from the STEP BD project, Goldberg et al. (2009) found that 67% of bipolar subjects were on two or more psychotropics, 40% of subjects received three or more psychotropic medications, while 18% of patients were taking four or more. Several international practice guidelines also recommend the optimal use of combination therapy to control both manic and depressive episodes in bipolar disorder (APA 2002; Yatham et al. 2009). Combining a mood stabiliser with an AAP may have therapeutic benefits, but these must be weighed against the additional side effect burden. These include extrapyramidal side effects and long-term metabolic and endocrine abnormalities. As Liauw and McIntyre (2010) comment, AAPs may evoke a different array of treatment-emergent adverse events than conventional agents; however, they do offer an improved therapeutic index when compared to conventional pharmacotherapies in bipolar disorder. A further clinical consideration involves the potential pharmacokinetic interaction which can occur between antiepileptics and second-generation antipsychotics such as aripiprazole (de Leon et al. 2012). Additional human studies and post-market surveillance are advised to clarify the safety of combining aripiprazole with antiepileptics such as carbamazepine.

In several double-blind randomised controlled studies of acute mania, combinations of lithium or divalproex with the AAPs risperidone, quetiapine, olanzapine and aripiprazole have demonstrated significant beneficial effects compared with lithium or divalproex monotherapy. In fact, for severe manic states, combination therapy is recommended as a first-line treatment (Yatham et al. 2009; Ketter 2008). For maintenance treatment in bipolar disorder patients who responded acutely to the addition of quetiapine to lithium or divalproex, continuing this combination reduced the subsequent risk of relapse to depression, mania or mixed states compared to lithium or divalproex alone (Suppes et al. 2009). Comparable data, however, are not available for combination with other AAP (Yatham et al. 2005).

In bipolar depressed patients, the evidence for added benefit from combination therapy with AAP has not been well studied. There is only one small double-blind randomised controlled study of aripiprazole as an augmentation agent in bipolar disorder. Quante et al. (2010) investigated 23 inpatients with Diagnostic and Statistical Manual of Mental Disorders-4th Edition (DSM-IV)-diagnosed bipolar major depression (Hamilton Depression Rating scale (HAM-D) ≥ 20) with a pre-existing stable regimen of lithium or valproate for at least 1 week. After inclusion, all patients were given adjunctive citalopram and either aripiprazole (*n* = 12) or placebo (*n* = 12) for 6 weeks. After 6 weeks of treatment, both groups had a significant reduction of HAM-D-rated depression of −18.71 for aripiprazole and −15.33 for placebo; however, this was not statistically significant between the groups. A noted deficit of this pilot study was the small sample, and as both groups responded well to citalopram, there was little chance for augmentation with aripiprazole to provide greater efficacy.

A common ongoing cause of suffering and disability in bipolar disorder is residual symptoms, usually either minor or subsyndromal depression (Strakowski 2007); it is for these symptoms that augmentation with another agent such an AAP is often attempted but for which there is virtually no data. Aripiprazole has a unique pharmacological mode of action that differs from current antipsychotics. It is a partial agonist that modulates dopamine activity (Yatham et al. 2005). Aripiprazole acts as a functional antagonist in a hyperdopaminergic environment and a functional agonist in a hypodopaminergic environment (Keck and McElroy 2003). There is evidence that bipolar disorder is associated with an alteration of the dopaminergic system, with increased dopamine function in mania and deficient function in depression (Keck and McElroy 2003). As a partial D2 receptor agonist, aripiprazole may therefore be well placed to correct both a functional deficiency and overactivity. Moreover, aripiprazole is also an antagonist at serotonin 5-HT2A receptors, a partial agonist at 5-HT1A receptors and an inhibitor of the 5-HT transporter (Yatham et al. 2005). These properties of aripiprazole have been hypothesised to mediate its putative antidepressant effects.

There is a need for further studies of AAP as combination or augmentation agents as they are more broadly being used in bipolar disorders. Therefore, the aim of this study was to evaluate the effectiveness of aripiprazole augmentation of maintenance treatment for bipolar disorder in patients with minor or subsyndromal mood episodes while on a stable dose of mood stabiliser and/or antidepressant.

## Methods

All subjects were outpatients, aged 18 years and above who had a diagnosis of bipolar I or II disorder confirmed by the Structured Clinical Interview for DSM-IV Axis I Disorders - Clinician Version. Patients were experiencing ongoing clinically significant symptoms but did not meet the full DSM-IV TR criteria for either major depressive episode or mania or mixed episode. All patients had been on a stable dose of a mood stabiliser and/or antidepressant at an adequate dose for at least 8 weeks prior to study entry. At baseline, all patients were interviewed and examined by a psychiatrist (IS or CN) to determine the diagnosis and suitability for entry to the study. Two research assistants who were trained in the use of the scales and who were tested for inter-rater reliability were used to assess the patients. All female patients of childbearing potential needed to use a medically accepted means of contraception. All participants provided written informed consent and were able to understand and comply with the requirements of the study. The study was approved by the Research and Ethics Committee at The Melbourne Clinic.

Patients who met one or more of the following criteria at enrolment were excluded from the study: (1) use of antipsychotic medication within 30 days of commencement of study, (2) use of depot antipsychotics during the last 8 weeks, (3) received ECT within the past 6 months, (4) current pregnancy or lactation, (5) current DSM-IV substance or alcohol dependence, (6) unstable or inadequately treated clinically significant medical illness, (7) current DSM-IV TR psychiatric diagnosis other than bipolar disorder and (8) significant risk for suicide.

Aripiprazole was administered in an open-label manner at a starting dose of 5 to 15 mg/day at baseline. After 2 weeks, the dose could be increased by 5 mg/day, then by a further 5 mg/day at week 4 and a further 5 mg/day at week 6, to a maximum of 30 mg/day, as clinically indicated. After week 8 of the study period, a 6-month extension period was permitted for those who responded to the treatment provided they agreed to ongoing two monthly assessments. At completion of the study or early termination, the dose was down-titrated if on more than 15 mg/day. The dose was reduced by 10 mg/day over a week.

Patients were assessed at weeks 0, 2, 4 and 8 with the Montgomery-Asberg Depression Rating Scale (MADRS), Young Mania Rating Scale (YMRS) and Clinical Global Impression of Severity (CGI-S). Tolerability was assessed using the Simpson and Angus scale (SAS), the Barnes Akathisia scale (BAS), the Abnormal Involuntary Movements scale (AIMS) and reports of adverse events. The changes in MADRS, YMRS and CGI-S scores from baseline to week 8 were analysed using one-way repeated measures analysis of variance (ANOVA) and last observation carried forward analysis. Responders as defined by a ≥50% decrease in MADRS and remission as a MADRS score of ≤8 were calculated.

## Results

Twenty-one patients entered the study, and follow-up ratings were available for 20 subjects. Patient demographics and clinical features at baseline are listed in Table 1. Seventeen (85%) patients completed week 4, while 14 (70%) patients completed 8 weeks. There were three (15%) patients taking one or more mood stabiliser (carbamazepine, lithium, valproate), 12 (60%) patients taking both a mood stabiliser (carbamazepine, lamotrigine, lithium, valproate) and an antidepressant (escitalopram, fluvoxamine, mirtazepine, moclobemide, tranylcypromine, sertraline, venlafaxine), while five (25%) patients were taking only an antidepressant (fluvoxamine, tranylcypromine, sertraline, venlafaxine).

**Table 1:** **Baseline demographics and clinical features of patients in study**

Patient demographics and clinical features	
Bipolar diagnosis, *n* (%)	
Bipolar I	6 (30)
Bipolar II	14 (70)
Females, *n* (%)	10 (50)
Caucasians, *n* (%)	20 (100)
Age (years), mean (SD)	47.9 (12.4)
MADRS, mean (SD)	20.5 (7.8)
Range	6 to 36
YMRS, mean (SD)	7.5 (6.5)
CGI-S, mean (SD)	4.2 (0.7)
Range	2 to 5
Weight (kg), mean (SD)	89.6 (19.6)
Starting dose (mg), mean (SD)	8.8 (3.2)
Range	5 to 15
Endpoint dose (mg), mean (SD)	11.5 (4.6)
Range	5 to 20

**Figure 1:**
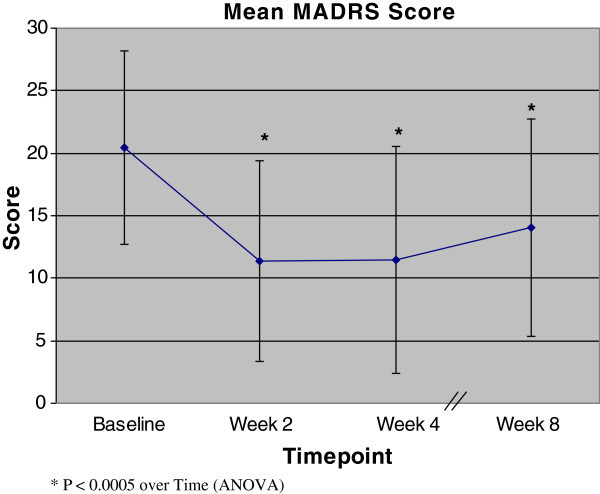
**MADRS scores (means ± SD) from baseline to week 8.** An asterisk indicates *p* < 0.0005 over time (ANOVA).

There was a significant decrease in MADRS scores over the course of treatment (*F* = 11.17; *df* = 3, 17; *p* < 0.0005). The improvement from baseline was significant at week 2 and remained so through to week 8 (Figure 1). On intention-to-treat at study endpoint, pooled MADRS scores reduced from 20.45(±7.78) to 14.05(±8.68), with a reduction of 6.40 points. Similarly, CGI-S scores significantly decreased over the course of study (*F* = 15.84; *df* = 3, 17; *p* < 0.0005), and again, this was significant from week 2 through to week 8. No correlation was found between the dose of aripiprazole and reduction of MADRS or CGI-S scores. There was no significant difference in YMRS scores over the study. Observed case analysis resulted in similar findings. Eight (40%) patients met MADRS response criteria while seven (35%) patients were classified as MADRS remitters at the last evaluation, whereas seven (50%) of the 14 patients who completed 8 weeks were classified as both responders and remitters.

When the data for the 14 bipolar II patients were examined separately, there was a significant improvement in MADRS scores over the course of treatment (*F* =7.39; *df* = 3, 11; *p* = 0.006). Improvement from baseline was significant at week 2 and week 4, but not at week 8, with a reduction of −5.07 on the MADRS from baseline (*p* = 0.058). CGI-S was similarly significantly improved from baseline to weeks 2 and 4, but not week 8. There was no significant improvement for YMRS at any of the time points. Response and remission rates were slightly lower in the bipolar II group only.

One subject withdrew before week 2 due to flu-like symptoms with no follow-up ratings. Three patients discontinued their treatment before week 4 and a further three before week 8 due to akathisia (1), agitation and anxiety (1), mild hypomanic symptoms (2) and inadequate efficacy (2). There were no significant differences in SAS, BAS or AIMS scores from baseline to week 8 either for the whole group or for the bipolar II group only. Nine patients continued with aripiprazole following the acute study. Two patients discontinued within the first 2 months due to relapse, five maintained their improvement for 6 months, and a further two patients continued for 11 to 12 months.

## Discussion

Aripiprazole was shown to be a modestly effective augmentation therapy for depressive symptoms in bipolar I and II disorders in this small open-label study. The patients in this sample did not meet DSM-IV criteria for a major depressive episode or mania or hypomania but were on stable maintenance medications with suboptimal mood symptom control. It is notable that aripiprazole was able to provide some improvement in this sample that were nonetheless significantly depressed. In our overall analysis, augmentation with aripiprazole did not significantly lower YMRS from week 0 to 8, but this is most likely due to the low mean YMRS of our patients at baseline. However, there were four patients with high YMRS at baseline (mean YMRS 19, range 16 to 22), and all were improved at week 8 with a mean YMRS of 3 (range of 0 to 4).

There have been two large double-blind randomised placebo-controlled trials of aripiprazole monotherapy in patients suffering from bipolar I and experiencing a major depressive episode (Thase et al. 2008). In both of these studies, aripiprazole monotherapy was no more effective than placebo at end point (week 8). The aripiprazole-treated patients were significantly more improved in the MADRS from weeks 1 to 6 for one study and for weeks 1 to 3 and 5, but separation from placebo was lost in the later weeks. The authors speculated that the dosing regimen may have been too high (starting dose of 10 mg) or titration was too rapid (5-mg increments per week) for this patient group, resulting in unexpectedly high rates of discontinuation. A meta-analysis of these RCTs (*n* = 690) (Fountoulakis et al. 2011), however, revealed a significant difference at week 8 in favour of treatment over placebo (*p* = 0.038). Regardless, the weak effect size of *d* = 0.17 and small relative reduction of −1.12 in the MADRS point to a limited benefit for its use as a monotherapy in bipolar depression.

There have been six open-label case studies of aripiprazole augmentation in bipolar depression (Ketter et al. 2006; Kemp et al. 2007; McElroy et al. 2007; Dunn et al. 2008; Sokolski 2007; Mazza et al. 2008). Three of the studies were retrospective chart reviews of patients with treatment-resistant depression (Ketter et al. 2006; Kemp et al. 2007; Sokolski 2007), and all but one study included both bipolar I and II patients (Sokolski 2007). Three studies included patients on aripiprazole monotherapy (McElroy et al. 2007; Dunn et al. 2008; Mazza et al. 2008). All studies reported improvement in depressive symptoms regardless of whether patients were on monotherapy or augmentation with aripiprazole. Response rates were 42% to 65% for the prospective studies. The study reporting the highest percentage of improvement (65%) was the largest (*n* = 85) and the longest (16 weeks) (Mazza et al. 2008). The most troublesome side effect was akathisia, ranging from 21% to 42% of patients. Aripiprazole was well tolerated in this study perhaps because of the low doses used with a mean final dose of only 11.5 mg. Other open augmentation studies found higher rates of akathisia than this study, which may be due to the higher doses used.

## Conclusions

Our study was limited by having an uncontrolled open-label design and a small sample size, while the maintenance therapies were also not standardised. Further, as the sample was modest, we could not ascertain which medications (as monotherapies or combinations) were more effective in combination with aripiprazole. Regardless, this pilot study adds to the evidence base for the potential utility of aripiprazole in both bipolar I and II patients who have ongoing subsyndromal depressive symptoms rather than suffering from a major depressive episode. Minor and subsyndromal depressive episodes continue to be the most common symptoms affecting bipolar patients and lead to significant impoverishment of quality of life and disability (Judd et al. 2002).

The implications of this study for supporting longer-term adjunctive treatment of aripiprazole in bipolar disorder are unknown. As this study and previous adjunctive trials are typically conducted over 6- to 8-week periods, longer-term studies are required to establish whether effectiveness of using aripiprazole may continue in this population. Of note, in our study, the main effect was during the first 2 weeks, with the antidepressant effect slightly diminishing from week 4 to week 8. This reflects the conclusions of Cruz et al. (2012) who found that both aripiprazole trials reviewed failed on the primary efficacy measure after the first 6 weeks.

Ideally, this study should be replicated without the above confounding variables and methodological deficiencies. Even though AAPs will continue to have an important role in bipolar disorders, either in combination or as an augmentation agent, the conduct of rigorous studies may be hampered by the complex study designs and large samples required, with potentially untenable financial implications. Although our study provides a positive signal for aripiprazole as a potential efficacious augmenting agent, the evidence is not conclusive, and it is premature to apply clinical recommendation at this stage.

### Key points

The following are highlights from the study:

- Aripiprazole was shown to be a modestly effective augmentation therapy for depressive symptoms in bipolar I and II disorders in this small open-label study.
- Although our study provides a positive signal for aripiprazole as a potential efficacious augmenting agent, the evidence is not conclusive, and it is premature to apply clinical recommendation at this stage.
